# Gut Microbiota and Mitochondria: Health and Pathophysiological Aspects of Long COVID

**DOI:** 10.3390/ijms242417198

**Published:** 2023-12-06

**Authors:** Laura Marinela Ailioaie, Constantin Ailioaie, Gerhard Litscher

**Affiliations:** 1 Department of Medical Physics, Alexandru Ioan Cuza University, 11 Carol I Boulevard, 700506 Iasi, Romania; lauraailioaie@yahoo.com (L.M.A.); laserail_mail@yahoo.com (C.A.); 2 President of the International Society for Medical Laser Applications (ISLA Transcontinental), German Vice President of the German-Chinese Research Foundation (DCFG) for TCM, Honorary President of the European Federation of Acupuncture and Moxibustion Societies, Honorary Professor of China Beijing International Acupuncture Training Center, China Academy of Chinese Medical Sciences, Former Head of Two Research Units and the TCM Research Center at the Medical University of Graz, Auenbruggerplatz, 8036 Graz, Austria

**Keywords:** ACE2, dysbiosis, dysfunction, electron transport chain (ETC), infection, hyperinflammation, immunomodulation, long COVID, microbiome, PACS, quantum, SARS-CoV-2

## Abstract

The current understanding of long COVID (LC) is still limited. This review highlights key findings regarding the role of gut microbiota, mitochondria, and the main pathophysiological aspects of LC revealed by clinical studies, related to the complex interplay between infection, intestinal dysbiosis, dysfunctional mitochondria, and systemic inflammation generated in a vicious circle, reflecting the molecular and cellular processes from the “leaky gut” to the “leaky electron transport chain (ETC)” into a quantum leap. The heterogeneity of LC has hindered progress in deciphering all the pathophysiological mechanisms, and therefore, the approach must be multidisciplinary, with a special focus not only on symptomatic management but also on addressing the underlying health problems of the patients. It is imperative to further assess and validate the effects of COVID-19 and LC on the gut microbiome and their relationship to infections with other viral agents or pathogens. Further studies are needed to better understand LC and expand the interdisciplinary points of view that are required to accurately diagnose and effectively treat this heterogeneous condition. Given the ability of SARS-CoV-2 to induce autoimmunity in susceptible patients, they should be monitored for symptoms of autoimmune disease after contracting the viral infection. One question remains open, namely, whether the various vaccines developed to end the pandemic will also induce autoimmunity. Recent data highlighted in this review have revealed that the persistence of SARS-CoV-2 and dysfunctional mitochondria in organs such as the heart and, to a lesser extent, the kidneys, liver, and lymph nodes, long after the organism has been able to clear the virus from the lungs, could be an explanation for LC.

## 1. Introduction

SARS-CoV-2 virus, a novel single-stranded ribonucleic acid (RNA) beta-coronavirus, belonging to the subgenus Sarbecovirus (beta-CoV lineage B), associated with a severe acute respiratory syndrome (Severe Acute Respiratory Syndrome Coronavirus), caused the 2019 coronavirus disease (COVID-19), declared a pandemic by the World Health Organization (WHO) from 11 March 2020, and which affected, to 2 August 2023, a number of 768,983,095 people, of which 6,953,743 people died worldwide; in Europe, out of the 3381 million cases, 53 million people died being confirmed with COVID-19, as reported by WHO. At the start of the pandemic, WHO announced “deep concern about the alarming levels of spread, severity, and inaction” and called on all countries to act quickly to prevent the spread of the virus. There were no effective treatment strategies, and the mortality rate was increasing, especially in patients infected with SARS-CoV-2 who also had comorbidities [1,2,3,4,5,6,7].

The human cost of COVID-19 for Chinese society, where the pandemic began in 2019, was very important but similar to that of the rest of the world. At the start of the pandemic, the loss of life was devastating for the elderly and vulnerable, while the social costs were, and still are, impossible to measure [8,9,10,11,12].

Clinical symptoms of the COVID-19 disease had a diverse spectrum, but in most cases, they were mild to moderate, and only in less than 5% of cases, critical disease phenotypes were manifested by disseminated coagulopathy, multiorgan failure, shock, and even death [13,14,15,16].

Clinical manifestations in patients with COVID-19 usually resolve within 2–3 weeks, but since the beginning of the pandemic, cases with persistent symptoms of chronic fatigue and post-exercise fatigue, digestive and respiratory disorders, cognitive dysfunction, and others have been observed, and some patients have developed new symptoms. All these manifestations have received different names, such as long COVID or LC, ongoing COVID, post-COVID-19 syndrome, chronic COVID-19 syndrome, post-acute COVID-19, and post-acute sequelae of COVID-19 (PASC) [17,18,19,20,21,22,23,24].

This situation was analyzed by WHO experts, and on 6 October 2021, through a Delphi consensus, they cataloged it in the clinical case definition of the post-COVID-19 condition, which includes the following: “post-COVID-19 condition occurs in people with a history of probable or confirmed SARS-CoV-2 infection, usually 3 months after the onset of symptoms of COVID-19 and lasting at least 2 months and cannot be explained by an alternative diagnosis. Common symptoms include fatigue, shortness of breath, cognitive dysfunction, and more, and generally impact daily functioning. Symptoms may have a new onset after initial recovery from an acute episode of COVID-19 or may persist after the initial illness. Symptoms may also fluctuate or recur over time” [25].

The WHO definition does not take into account the considerable diversity of symptoms within the same group and the different manifestations of the post-COVID-19 condition, and for this reason, many researchers prefer the LC term. Grouping symptoms into categories of phenotypes or clinical syndromes of LC that would possess the same pathophysiological mechanisms is the focus of some current studies [26,27].

Depending on the size of the studied cohort, the data collection methods, the clinical symptoms entered, and the methods of analysis, several heterogeneous phenotypes of LC can be distinguished. The statistical diagnosis that is reported in the United States is U09.9, the ICD-10-CM code of “post-COVID-19, unspecified condition” with the highest frequency, and algorithmically includes four large categories of conditions: cardiopulmonary, neurological, gastrointestinal, and comorbidities. Statistical studies using unsupervised clustering revealed the most common LC cardiorespiratory phenotype, in which patients complain primarily of respiratory problems, while chest discomfort, shortness of breath, and palpitations are present to varying degrees [28,29,30,31].

Some authors believe that patients who have previously suffered a severe acute infection with hospitalization and major organ damage or dysfunction may be included in the non-syndromic LC phenotype and may respond well to traditional means of rehabilitation. Another phenotypic category of LC, considered LC syndrome, could include patients who previously had a mild acute infection without hospitalization but who were then left with extreme fatigue, chest pain, cough, breath difficulties, shortness of breath, chest tightness, polypnea, and decreased exercise tolerance. Typically, this subtype of LC patients has a history of comorbidities (e.g., myalgic encephalomyelitis/chronic fatigue syndrome (ME/CFS)) that have been aggravated by the acute infection and do not respond well to conventional therapeutic methods. Another subtype of LC would be secondary acute COVID-19, in which the clinical symptoms gradually disappear over several months without any treatment [32,33,34,35,36,37].

It is estimated that around 100 million people worldwide have LC, for which diagnosis, prognosis, and treatment options are still limited [38]. The prevalence of COVID-19 symptoms varies from country to country, according to reports published by different authors. Some studies mention that after two months of infection with COVID-19, about 87% of patients still have at least one symptom, 32% have one or two symptoms, 55% have three or more symptoms, and 50% of patients meet the criteria for ME/CFS, and most have symptoms that worsen after exercise. The long-term consequences of the SARS-CoV-2 infection result in huge healthcare costs for recovery, and the economic burden on medical rehabilitation centers is not yet precisely known. In the United States alone, fund managers have estimated that a post-COVID recovery will require at least $4.4 trillion in the future [39,40,41,42,43,44,45,46].

## 2. Molecular and Cellular Pathophysiological Mechanisms at Gut Level in Long COVID

The pathophysiology of LC is a hot, unresolved, current topic that raises the opinions of many experts who have proposed various scenarios, but which generally have roughly the same guidelines. Although the intestine and the lung apparently function separately, these two organs share the same embryonic origin and similar morphological components. In the fetal period, starting at about ^th^e 4th week, the lung bud develops as a protrusion of endodermal tissue from the foregut and then forms the laryngotracheal bud. After the 16th week and throughout infancy, the lung continues to develop and mature [47].

Comparing the structures of the lung with those of the intestine, it can be noticed that both are covered with mucous membranes that produce a nitrogenous glycoprotein called mucin, which has a local protective role and a common immunological ability to defend through the mucosa. There is evidence to show that when changes occur in the gut microbiota, signals are also transmitted to the lungs. Thus, gut microbiota and microbial metabolites actively participate in monitoring and regulating lung microbiota composition in cases of inflammation or infection and may even reject lung transplantation [48,49].

The entry of the SARS-CoV-2 virus into the human body will change the microbiota and the balance of microbial metabolites, which will lead to important systemic disorders with organ damage and the appearance of a variety of clinical symptoms. The gut microbiota has been shown to contribute to the regulation of angiotensin-converting enzyme 2 (ACE2) expression in the renin-angiotensin complex through systemic and local pathways. ACE2 is already known to be the cornerstone of SARS-CoV-2 infection in the COVID-19 disease due to the specific coupling of the spike protein of the SARS-CoV-2 virus. While in the intestine, particularly at the “brush” border of proximal and distal enterocytes in the small intestine, ACE2 expression is more than fourfold higher than in other tissues and is reduced in the lung [50], which makes it plausible to consider the intestine as the entry point of the virus into the human body.

Because of research gaps and especially the fluctuation of current hypotheses in the literature, numerous studies are needed to better understand the relationships between the gastrointestinal tract and its involvement in LC, to better manage the disease, and to discover innovative treatments or means of preventive intervention at the digestive tract level [51].

The pathophysiological processes leading to the appearance of the more than 200 symptoms of LC are believed to have a multifactorial etiology that includes host conditions (age, sex, ethnicity, genetic factors, metabolic or endocrine diseases, chronic inflammation, immunological imbalances, and autoimmune diseases), viral agents (occult persistence of SARS-CoV-2 or its viral components and reactivation of latent viruses), and downstream effects (grade of lesions from primary acute SARS-CoV-2 infection, vascular endothelial abnormalities, microclots, thrombosis, neurological signaling dysfunction, reduction in tissue oxygen, and disruption of the gut microbiome) [37,52,53,54,55] (Table 1).

In relation to the immune response to SARS-CoV-2 infection and inflammatory diseases, current research mentions that there are significant differences between the two sexes. The female sex is stronger in the face of viral infection due to the special nature of innate immunity, steroid hormones, and factors related to sex chromosomes. The two X chromosomes in females led to greater immune responses that increased resistance to viral infections, correlated with increased toll-like receptor 7 (TLR7), interferon (IFN) production, antiviral antibodies, and the generation of pro-inflammatory cytokines of the interleukin-6 and -1 family (IL-6 and IL-1). The extra X chromosome exerts a stronger influence on the immune system, and various molecules, including toll-like receptor 8 (TLR8), CD154 (CD40 or CD40L ligand), and CX3C-motif chemokine receptor 3 (CXCR3), appear overexpressed in females, with favorable consequences regarding a good response to viral infections and vaccinations. However, biallelic expression of X-linked genes can promote autoimmune responses and harmful inflammatory diseases. These hypotheses come to motivate the much higher prevalence of LC in the female sex, as described in all published studies [56,57,58,59].

The mentioned aspects regarding the relationship between age and the risk of developing LC need to be studied for a long time to answer the complex issues related to the conjunction between advanced age, senescence of the immune system, specific biological systems, and clinical phenotypes [60,61,62].

Studies published in the United States (US) and the United Kingdom (UK) highlight higher risks for black, Afro-Caribbean, Polynesian, Middle Eastern, or Native American patients to have more severe forms of COVID-19 and LC symptoms compared to the Caucasian population. The inexplicable problem is whether these aspects are related to the quality of medical care, treatments received, reports, or whether ethnicity is really involved [63,64,65].

Many genetic studies are still needed to uncover risk factors within human leukocyte antigen (HLA) molecules and to establish the relationship between genetic susceptibility to SARS-CoV-2 infection, its severity, association with an increased risk of LC, and confidence for future vaccination strategies [55,66,67,68].

If the involvement of ethnicity and genetic factors is not fully elucidated, instead it has been shown that the persistence of the SARS-CoV-2 virus, its spike protein, and viral RNA in the occult spaces of the brain, sinuses, adrenals, kidneys, intestine, ganglia lymphatics, spleen, lungs, and even the heart is in about 30% of LC patients. The persistence of the virus and its components a year or more after the acute infection with SARS-CoV-2 brings with it clinical manifestations secondary to local inflammatory phenomena, the stimulation of autoimmune processes, and the triggering of an aberrant immune response that leads to an accelerated state of inflammation, which then becomes chronic and sometimes intervenes directly through its cytopathic action. Another interesting aspect of the mode of action of SARS-CoV-2 is the persuasive stimulation of the immune system until the exhaustion of T cells, reducing the potential for viral elimination, together with the phenomenon of molecular mimicry of viral self-proteins to evade the surveillance of the immune system and trigger a pathology with the characteristics of an autoimmune disease [69,70,71,72].

Increasingly recent studies suggest that excessive immunological responses may underlie the severity of the initial clinical manifestations of SARS-CoV-2 infection as well as the subsequent symptoms of LC. The multivalence of innate and adaptive immune system reactions in the acute phase and later in the post-acute period of the disease requires deep investigations to uncover the specific molecular pathways as well as the categories of immune cells involved in the pathophysiology of LC [73].

In individuals with LC, prolonged T cell activation time with increased expression of activation or exhaustion markers, such as human programmed cell death protein 1 (PD-1) and T cell immunoreceptor with Ig and immunoreceptor tyrosine-based inhibitory motif (ITIM) domains (TIGIT: T-cell immunoreceptor with immunoglobulin and immunoreceptor tyrosine-based inhibitory motif domains) [74], a decrease in the number of naïve B lymphocytes, and increased activation of innate immune system cells and myeloid cells are concomitant with the increased secretion of the production of type I and type III interferons [75,76].

Impairment of PD-1 functions has serious consequences for host defense mechanisms, as this surface receptor protein plays a major role at the immune checkpoint by regulating T-lymphocyte activity during immunity and tolerance. Through the interactions of PD-1 with its ligands PD-L1 and PD-L2, protection against autoimmunity phenomena is ensured by supporting the apoptosis of antigen-specific T lymphocytes in the lymph nodes and reducing the apoptosis of regulatory T cells. The modulation of the PD-1/PD-L1 pathway (by inhibiting the interaction between PD-1 and PD-L1) has been shown to increase T-cell activity with beneficial effects in cancer control and cure using monoclonal antibodies (e.g., IgG4) [77,78,79,80].

It has been shown that many other circulating cytokines, including IL-6, IL-8, IL-10, tumor necrosis factor-alpha (TNFα), transforming growth factor-β (TGFβ), and IFN-gamma-inducible protein 10 (IP-10 and CXCL10), have increased levels in patients with various LC symptoms [81,82,83,84].

Due to a phenomenon of hyperactivity of the immune system and the excessive formation of autoantibodies after infection with SARS-CoV-2, we can suspect the occurrence of autoimmune diseases, such as multiple sclerosis, Guillain-Barré syndrome, Kawasaki disease, various types of vasculitis, thrombocytopenia, hemolytic anemia, prothrombotic state, diffuse coagulopathy, etc. Autoimmunity is a common feature in some LC phenotypes, possibly due to the persistence of viral antigen, an inadequate response, or the intervention of factors in host tissue immune regulation. The prevalence of autoantibodies in people with LC symptoms is much higher than in the general population. Latent autoimmunity has been shown to correlate directly with the humoral response to SARS-CoV-2. To date, six categories of autoantibodies have been detected against structures of the cardiovascular system, thyroid gland, components of the immune system, G protein-coupled receptors specific for rheumatic diseases, and other autoantibodies, highlighted in COVID-19; however, the presence of these autoantibodies has not yet been shown to be pathogenic and clearly correlated with genetic predispositions, sex, age, and clinical symptoms [85,86,87,88].

## 3. Gut Microbiota Dynamics in Long COVID

A growing number of studies have shown that the gut bacterial microbiome of patients with COVID-19 was greatly altered compared to healthy people, in that there was a significant reduction in commensal bacteria, which were replaced by opportunistic pathogens. In healthy individuals, the taxa *Eubacterium*, *Faecalibacterium prausnitzii*, *Roseburia*, and *Lachnospiraceae* form an integral and predominant part of the natural gut microbiome, whereas in patients infected with SARS-CoV-2, their feces were enriched with opportunists, which include *Clostridium hathewayi*, *Actinomyces viscosus*, and *Bacteroides nordii*. Dysbiosis of the gut microbiome that occurred during the manifestations of COVID-19 has been observed to persist even in patients who have recovered from the disease, and changes in the microbiota could underlie chronic symptoms, demonstrating that the gut microbiome is directly correlated with the condition of host health in the LC phase [89,90,91].

The first studies related to the identification of the taxonomic structure of the intestinal microbiota were carried out by Liu et al. in LC. In this study, 106 patients with varying degrees of severity of COVID-19 after 6 months of disease onset were prospectively investigated, compared to 68 non-COVID-19 patients. In total, 258 stool samples were analyzed using metagenomic sequencing for correlation with persistent symptoms. After 6 months, 76% of patients had LC symptoms (e.g., fatigue, poor memory, and hair loss) and a microbiome with increased levels of *Ruminococcus gnavus*, *Bacteroides vulgatus*, and lower levels of *Faecalibacterium prausnitzii* compared to the normal appearance of the control group. Respiratory manifestations and neuropsychiatric symptoms correlated directly proportionally with nosocomial intestinal pathogens such as *Clostridium innocuum* and *Actinomyces naeslundii* and inversely proportionally with *Bifidobacterium pseudocatenulatum* and *Faecalibacterium prausnitzii*—butyrate-producing bacteria [92].

Mazzarelli et al. investigated the gut microbiota in a cohort of 97 patients with mild or severe SARS-CoV-2 infection (47 patients with a good prognosis and 50 patients with a “severe” prognosis). Gut microbiota composition was studied from rectal swabs by sequencing the V3–V4 hypervariable region of the 16S rRNA gene and PEnalized logistic regression analysis (PELORA). In patients with more severe forms of respiratory failure, the phyla Campylobacterota and Actinobacteriota were found in a much higher percentage [93].

Changes in the intestinal microbiota in the sense of dysbiosis can underlie the disruption of neuronal structure and function, but also some behavioral changes (for example, autism, anxiety, depressive disorder, and schizophrenia) on the microbiome–gut–brain axis, especially in patients with LC. Remodeling the gut microbiota with food and/or probiotics, such as, for example, *Lactobacillus rhamnosus* (ATCC 53103), *Lactobacillus helveticus* R0052, *Bifidobacterium longum* R0175, *Saccharomyces boulardii*, and *Lactobacillus casei Shirota*, significantly improved stressful behaviors [94,95].

Gao et al. investigated the effects of stressful activities on mental health and gut microbiota in 71 first-line healthcare workers (FHWs) who treated patients with COVID-19 for two months in isolation wards in Wuhan, China, compared with 104 healthcare workers from the second-line healthcare workers (SHWs), who treated uninfected patients with COVID-19 routinely admitted to hospitals. The research was carried out at four different times as follows: immediately after the end of their shift (day = 0), after a two-week quarantine in a hotel (day 14), four weeks after returning to normal life (day 45), and half a year after working on the front line (day 180). The authors used full-length 16S rRNA gene sequencing for bacteria in the microbiota and analyzed the influence of microbial changes on the mental status of FHWs. The authors observed that stressful activities promoted significant neuropsychiatric symptoms such as depression and anxiety in FHWs and produced intestinal dysbiosis for a period of several months. At the same time, *Faecalibacterium* Spp. and *Eubacterium eligens group* spp., bacteria in the group of 15 “good gut microbes” that produce butyrate, “healthy” fats, and anti-inflammatory power by stimulating the production of IL-10, were reduced. *Eubacterium hallii* bacteria with an important role in the intestinal metabolic balance decreased, and potentially pathogenic microbes from the genus Bacteroides increased in abundance. Stressful activities during the therapy of patients with COVID-19-induced gut dysbiosis have negative effects on mental health in FHWs [96].

Although patients with COVID-19 mainly present symptoms of respiratory infection, many studies demonstrate the involvement of the gastrointestinal tract in the severity of the disease through intestinal barrier dysfunction and dysbiosis. In a randomized trial of 181 hospitalized patients with COVID-19, of whom 149 completed the 3-month follow-up, Vestad et al. investigated pulmonary function tests in 108 patients and gut microbiota in 97 patients from rectal swab material and then by 16S rRNA gene sequencing. The results showed that patients with COVID-19 and respiratory function disorders had an impoverishment in the complex structure of the intestinal flora, with the presence of *Veillonella*-type taxa, possibly involved in pulmonary fibrosis. At the same time, a strong correlation was observed between high plasma concentrations of lipopolysaccharide-binding protein (LBP) and respiratory dysfunction, aspects that persisted beyond 3 months after discharge. In conclusion, the reduced microbial diversity and structural changes in the gut microbiota in patients with respiratory dysfunction after 3 months, as well as the association of persistently elevated LBP levels with clinical impairment of respiratory function, indicate the potential involvement of the gut on the lung axis in COVID-19. These observations could be related not only to acute respiratory failure during hospitalization but also to morbidity in LC. This study has its limitations and should be considered exploratory [97].

Liu, Q. et al. undertook a study of 133 hospitalized patients with COVID-19 between 13 March 2020 and 27 January 2021. They investigated viral RNA concentrations in nasopharyngeal swabs and stool samples using quantitative real-time reverse transcription-PCR (RT-qPCR). At the same time, they investigated the serum levels of cytokines and chemokines and the different types of mononuclear cells in freshly collected peripheral blood. Simultaneously, the structure of the intestinal microbiome was studied in 296 fecal metagenomes and 79 fecal metabolomics at three longitudinal time points after hospitalization. From the respiratory tract, the viral load was analyzed in 1378 samples and correlated with the clinical symptoms of the 133 patients with COVID-19 who were prospectively monitored for 6 months. Metagenomic analysis of the gut multibiome (bacteria, fungi, and viruses) revealed two robust ecological clusters (Clusters 1 and 2). In Cluster 1 patients, the multibiome structure contained a reduced biome diversity compared to Cluster 2, and the bacteria *Ruminococcus gnavus* and *Klebsiella quasipneumoniae*; fungi such as *Aspergillus flavus*, *Candida glabrata*, and *Candida albicans;* and viruses such as *Mycobacterium phage MyraDee* and *Pseudomonas virus Pf1* predominated. Also, when examining upper respiratory tract samples and fecal samples, a longer period of viral positivity was observed in Cluster 1 compared to samples from Cluster 2 patients. Clinical manifestations (fever, chills, diarrhea, cough, etc.) and biological parameters (e.g., C-reactive protein (CRP) and CXCL10) in cohort 1 patients were strongly associated with severe COVID-19 and, in addition, with the occurrence of LC. The authors support the utility of investigating host phenotype and gut multibiome profiling as a prognostic tool in patients with COVID-19 [98].

The structure of the gut microbiome is imprinted by previous infections and is closely related to immunity, inflammation, and the severity of the COVID-19 infection. Tkacheva et al., using the 16S rRNA sequencing technique on stool samples, investigated the gut microbiome in 178 patients with post-COVID-19 and contacts for SARS-CoV-2, but without infection. This batch of patients was divided into three groups: asymptomatic (n = 48), COVID-19 contacts without other infections (n = 46), and patients with severe COVID-19. Using a new compositional statistical algorithm for cardiovascular clinical parameters, immunity, markers of endothelial dysfunction, blood metabolites, and the concept of co-occurring bacterial clusters (coops), the results showed that although clinically the parameters varied drastically between groups, no differences in gut microbiome characteristics were found. The results of the research demonstrated that the intestinal microbiota has the ability to recover after SARS-CoV-2 infection, and therefore, after three months, no significant differences were found in its composition in patients with different degrees of LC severity [99].

Many data in the literature provide evidence of the direct participation of the gastrointestinal tract in the acute and late clinical manifestations of COVID-19. Aberrant immune activity during the clearance process of SARS-CoV-2 appears to induce severe disease symptoms and residual late inflammatory activity. In this context, Caio et al. investigated whether SARS-CoV-2 infection can be correlated with disturbances in the composition of the intestinal microbiome, with the severity of clinical manifestations, and with the consequences of the disease. In total, 46 patients, aged between 30 and 95 years, hospitalized with COVID-19, from whom stool samples were analyzed by shotgun metagenomic sequencing, were studied. To detect the effects of disturbances in the composition of the intestinal microbiome, the authors grouped the patients according to the degree of severity of clinical signs (12 patients were critical and 34 non-critical patients), the place where they were hospitalized (intensive care or non-intensive), and the final outcome (survival or death). The results showed that the severity of clinical symptoms of COVID-19 was directly proportional to the changes in the gut microbiome, the decrease in microbial biodiversity, and the abundance of the *Escherichia* and *Bacteroides genera*. The extent of dysbiosis did not correlate with the severity of clinical manifestations. An increase in microeukaryotic species (*Candida albicans*, *Candida tropicalis*, and *Saccharomyces cerevisiae*) and unhealthy cardiometabolic markers (*Escherichia coli*, *Bacteroides fragilis*, *Clostridium bolteae*, *Clostridium innocuum*, *Clostridium symbiosum*, *Eggerthella lenta*, *Enterococcus faecium*, and *Flavonifractor plautii*) were detected. Reconstitution of the SARS-CoV-2 genome from patient fecal samples confirmed the accuracy and sensitivity of the metatranscriptomic method for recognizing probable virus variants. The study suggests a correlation between the level of intestinal dysbiosis and the severity of the disease, probably through the elimination of microorganisms with an immunomodulatory effect. At the same time, intestinal dysbiosis would underlie the persistent inflammatory process and complications after the virus elimination process [100].

Zhang D. et al. conducted a prospective study to monitor symptoms and clinical indices of recovered patients (RPs) one year after discharge from Union Hospital, Wuhan, China, between December 2020 and May 2021. In this study, 16S rRNA sequencing of stool samples was performed in RPs and in healthy controls (HCs), and, at the same time, the relationship between gut microbiota and LC symptoms was studied. The research included 187 RPs, of whom 44.9% had clinical manifestations of LC one year after discharge. The predominant symptoms were cardiopulmonary (chest tightness after activity, palpitations on exertion, cough, sputum, and chest pain), followed by systemic manifestations such as fatigue, muscle pain, and gastrointestinal signs (anorexia, diarrhea, or constipation). A percentage of 35.9% of RPs presented neuropsychic signs such as anxiety or depression, which were significantly more frequent in symptomatic patients. From the total number of monitored subjects, 130 RPs and 32 HCs underwent the sequencing study of fecal samples. The symptomatic RPs group showed gut microbiota dysbiosis with a significant decrease in bacterial diversity and short-chain fatty acids (SCFAs) produced by *Eubacterium_hallii*, *Subdoligranulum*, *Ruminococcus*, *Dorea*, *Coprococcus*, and *Eubacterium_ventriosum*, compared to HCs. The authors provide evidence for the role of gut microbiota in maintaining long-term symptoms of COVID-19 for one year after discharge in RPs [101].

LC, or symptoms remaining long after the SARS-CoV-2 infection was gone, has caused worldwide concern as some patients lose certain abilities and their quality of life is greatly diminished. Zhang, D. et al. conducted research on the functional aspects of the oral and intestinal microbiome as well as serum metabolites in subjects with digestive manifestations who had LC. The authors prospectively analyzed oral, fecal, and serum samples from 983 antibiotic-naïve subjects with mild COVID-19 who were then monitored for 3 months after discharge. Here, 45 fecal and saliva samples and 25 matched serum samples were collected from subjects who had LC with digestive symptoms and, at the same time, from healthy controls (HCs). Then, 8 samples of feces and saliva were collected from patients with LC who did not present digestive symptoms. Shotgun metagenomic sequencing of fecal samples and 2bRAD-M sequencing of saliva samples were performed. Paired serum samples were studied for large-scale metabolomics. The results showed that subjects with mild forms of LC without long-term digestive symptoms of COVID-19 did not show a significant difference in gut and oral microbiota during hospitalization or later compared to HCs. In contrast, samples collected initially and after 3 months from patients with digestive symptoms in LC were significantly altered. Ectopic colonization of the oral cavity with intestinal microbes from the *Proteobacteria phylum* was also observed. The abundance of *Neisseria*, *Lautropia*, and *Agrobacterium microflora* was correlated with the potential toxicity of serum metabolites such as 4-chlorophenylacetic acid, 5-sulfoxymethylfurfural, and estradiol valerate. The study reveals that ectopically colonized bacteria from the gut in the oral cavity in LC patients with digestive symptoms were correlated with some potentially harmful serum metabolites and could underlie the pathogenic mechanisms of this pathology [102].

These analyzed studies are presented in Table 2.

## 4. Mitochondria and Long COVID—The Hidden Molecular Connections and the Quantum Leap

Over the past two decades, the role of mitochondrial function in health and disease has become increasingly recognized, and rapid advances in our scientific knowledge show tremendous promise to one day elucidate the mysteries of this ancient organelle to therapeutically modulate it when it is necessary. However, we still have much to clarify and understand about how viruses take control of infected cells to multiply, relying entirely on hijacking the host cell’s own molecular machinery, strategically influencing cellular metabolism, and altering not only the structure but also the functions of the organelles inside the cell, implicitly the mitochondria, which could be a key factor in the pathogenesis of COVID-19. Mitochondria are versatile and critical metabolic structures not only for energy production but also for modulating cellular immunity through several mechanisms, which generate cell apoptosis, reactive oxygen species (ROS) signaling, mitochondrial antiviral-signaling protein (MAVS) activation, and deoxyribonucleic acid (DNA)-dependent mitochondrial immune activation. These events are regulated by mitochondrial dynamics and its damage-associated molecular patterns (mtDAMPs), complex phenomena by which mitochondria change, including their length and connectivity (structure), in response to the stress generated by the virus that hijacks all processes to avoid the immune response mediated by mitochondria to survive and proliferate, thus generating even more cellular stress and inducing disease progression [103,104,105].

Although post-viral fatigue syndrome has been known for decades, the mechanism by which viruses cause this syndrome is still unknown, and it is assumed that the breakdown of mitochondrial metabolic pathways could be the main cause. Similarly, COVID-19 infection could produce a redox imbalance that causes long-term, the so-called LC. There is a bidirectional connection between redox dysregulation, systemic inflammation, the damaged ability to generate adenosine triphosphate, and the general hypometabolic condition. Comprehending all these phenomena and the molecular basis of LC may lead to innovative management modalities [106,107,108].

Grossini et al. investigated 60 elderly patients hospitalized in a long-term care (LTC) facility for at least 1 year. The levels of oxidants/antioxidants, cell viability, and mitochondrial function were determined through blood plasma analyses and correlated with clinical data for influenza-like illnesses and COVID-19. The follow-up proved a positive correlation between mitochondrial function and the clinical evolution of patients, a balanced redox state in most of the elderly, an approximately 20% decrease in the mitochondrial membrane potential of human umbilical vascular endothelial cells (HUVEC) in patients with flu-like symptoms, or even a confirmed diagnosis of COVID-19. Assessment of mitochondrial function in the elderly could provide important clues for their clinical management in LTC facilities [109].

It is recognized that sarcopenia is determined by inflammatory processes, oxidative stress in skeletal muscles, and mitochondrial dysfunctions, including the imbalance between the synthesis and degradation of muscle cells. Levy et al. included in a prospective study a group of 139 patients infected with SARS-CoV-2, starting from the time of hospitalization and 3 and 6 months after discharge, and studied the evolution of weight and sarcopenia using the criteria of the European Working Group on Sarcopenia in Older People (EWGSOP2), muscle strength, and mass using hand dynamometry and dual-energy X-ray absorptiometry. Applying multivariate analysis, the research highlighted that the only factor associated with sarcopenia is the duration of hospitalization in the intensive care unit (ICU). The weight dropped even more than 5–10% in the group with sarcopenia, but at 6 months, 16 of the 22 patients no longer had sarcopenia and had regained their normal previous weight. Sarcopenia and malnutrition could be present even 3 months after COVID-19, but for about two-thirds of patients, this condition could be reversed 6 months after discharge with nutritional support and personalized rehabilitation [110].

Ghanem et al. have systematically examined, over a longer period after discharge, the evolution of patients admitted to the ICU who were infected with SARS-CoV-2 and their subsequent evolution regarding the ability to be autonomous, concurrently with poor nutrition and/or defective digestion, as well as the other clues and signs if present. Thirty-seven patients with a mean age of 64.3 ± 14.3 years were included in the study and were analyzed concerning the quality of being autonomous applying the “Autonomie Gérontologie Groupes Iso-Ressources” (AG-GIR) classification; the nutritional status using the French and Global Leadership Initiative on Malnutrition (GLIM) recommendations; as well as their outcome starting with the moment prior to being infected, during the ICU and at admission to the rehabilitation center (CRM), and up to half a year after they returned to their residence. Before contacting the SARS-CoV-2 virus, all patients were autonomous, 39% of whom were non-autonomous upon admission to the CRM and required significant help. After the rehabilitation period, only 6% were not perfectly autonomous, and in another 6 months, the percentage was halved, that is, only 3% still needed considerable supervision and help. Regarding the state of nutrition, of the total number of patients studied, 81% had severe malnutrition, and only 11% had moderate malnutrition, proving an important correlation (*p* < 0.05) between the state of nutrition and the ability to be autonomous. The most important symptom that was persistently present in 70% of the subjects and half a year after discharge was chronic fatigue; 20% of the subjects did not regain full autonomy, while all other symptoms greatly diminished. The value of the study is that it highlights the correlation between autonomy and poor nutritional status after a severe form of COVID-19, the need for personalized and sustained recovery programs, and nutritional recommendations for patients to regain autonomy who, despite all these measures, may still suffer from chronic fatigue, even 6 months after discharge [111].

Changes in metabolism and mitochondrial impairment are revealed by exercise intolerance in LC patients. These post-acute sequelae relate to higher arterial blood lactate collection and decreased fatty acid oxidation rates during exercise tests. Guntur et al. analyzed the plasma metabolic profiles of 45 post-acute COVID-19 patients who were divided into two groups: G1 (n = 29) who developed LC and G2 (n = 16) who fully recovered, using mass spectrometry-based untargeted metabolomics, compared to G3 (n = 30) of healthy controls. The main symptoms listed by LC patients in G1 were fatigue, palpitations, chest discomfort, dyspnea, and brain fog. Plasma metabolic phenotypes in LC, when compared to fully recovered and healthy controls, revealed marked changes in fatty acid biosynthesis, activation, metabolism, beta-oxidation, and tricarboxylic acid cycle pathways. Impairment of fatty acid oxidation during induced exercise reflects lactate accumulation and inadequate respiratory gas exchange, suggesting mitochondrial dysfunction, which supports the hypothesis of mitochondrial dysfunction as the basis of metabolic manifestations in LC. The limitations of the study are the small samples [112].

Mitochondria, subcellular organelles with lots of important energetic roles, can affect the integrity and operationality of living cells, and their running can be rated by measuring the mitochondrial membrane potential (ΔΨm). It is well known that a decrease in ΔΨm correlates with several conditions triggered by inflammation, phenomena also present in the case of infection with SARS-CoV-2. Díaz-Resendiz et al. performed a comparative study of ΔΨm in human peripheral blood mononuclear cells (HPBMC) isolated from four groups as follows: group 1 (n = 35) or HC (healthy controls); group 2 (n = 36) or C-19, patients with COVID-19 (qRT-PCR positive); group 3 (n = 34) or R1, recovered subjects who returned after 40 ± 13 days for laboratory analysis; and R2 (n = 18), subjects returned after 335 ± 20 days after infection. Compared to healthy subjects in HC, ΔΨm significantly decreased in C-19, R1, and R2 groups, highlighting that it changes during infection and remains altered post-COVID and in LC. Of the 18 subjects presented in R2, 85% had LC-related symptoms (fatigue, insomnia, joint pain and arthralgia, headache and memory loss, anxiety, dry cough, chest pain, dyspnea, anosmia, and others), well correlated with the loss of ΔΨm still present at 335 ± 20 days post-infection [113].

Because fucoidan, a polysaccharide synthesized from brown seaweed, has been shown to have different biological effects, such as anti-inflammatory, antioxidant, anticoagulant, antibacterial, and antineoplastic, Díaz-Resendiz et al. investigated the influence of fucoidan (20 and 50 μg/mL) on the mitochondrial membrane potential (ΔΨm) of leukocytes from different groups of patients: those with active disease and those who recovered from infection, compared to healthy subjects. The research included 76 subjects with an average age of 40 years in the range of 18–64 years, namely with the following gender distribution: 30 men and 46 women, divided in the first phase as follows: group 1 = healthy control subjects (HC, n = 24), group 2 = COVID-19 patients (C-19, n = 31), and group 3 = recently recovered from COVID-19 (R1, n = 21), that is, 40 ± 13 days after infection. In phase 2, in order to report long-term sequelae or LC, ∆Ψm was also measured 11 months (335 ± 20 days) after infection with SARS-CoV-2 in the R2 group (n = 19) compared to HC (n = 19), healthy subjects. The experiment proved that fucoidan remarkably restored ∆Ψm of HPBMC, so this treatment would be useful to advance to a better state of mitochondrial homeostasis after COVID-19 [114].

To reveal the repercussions of SARS-CoV-2 infection on the metabolism of mitochondria and the possible connection with LC, Pozzi A. studied the expression of several mitochondrial non-coding RNAs in post-COVID-19 patients using published, ready-for-use sources. The author included plasma samples from 10 individuals positive for COVID-19, hospitalized and enrolled between January 2020 and April 2020, and 10 age-matched healthy controls. All RNA samples were extracted from peripheral blood mononuclear cells (PBMC). No disparity in the expression of long non-coding RNAs was identified during infection; only 43 small mitochondrial RNAs had altered expression during post-COVID-19 recovery [115].

Clinical experience has demonstrated that poor outcomes in patients with COVID-19 are related to systemic hyperinflammation and immunopathology. Lage et al. investigated whether the inflammasome and oxidative stress are independently involved in the severity of this pathology or whether the two pathways synergistically contribute to the worsening of the condition. Participants were enrolled in the so-called “CALYPSO” (COVID-19-associated Lymphopenia Pathogenesis Study in Blood) protocol, that is, blood samples from healthy volunteers or healthy control individuals (HCs) (who were de-identified before randomization). In this, patients were enrolled according to their highest oxygen requirement at the time of the research blood draw and classified into (1) mild cases (n = 31; 23 inpatients and 8 outpatients, respectively) with oxygen requirements of 4 L (l) or less, including no oxygen; (2) moderate cases (n = 4, oxygen concentration of up to 50%); and (3) severe cases (n = 12), where patients required more than 50% oxygen concentration or ICU care. The investigators grouped patients with moderate and severe forms of the disease into the same group, called moderate-severe (n = 16). The study included measurements of plasma inflammatory biomarkers, such as interferon-gamma (IFN-γ), interleukin (IL)-2, IL-6, IL-7, IL-8, IL-10, IL-12p70, IL-27, tumor necrosis factor-alpha (TNF-α), E-Selectin, P-Selectin, intercellular adhesion molecule (ICAM)-3, serum amyloid A (SAA), CRP, vascular cell adhesion molecule 1 (VCAM)-1, ICAM-1, and IL-18; ferritin heavy chain (ferritin-H); D-dimer; levels of iron; concentrations of catalase; superoxide dismutase activity (SOD); total oxidant status; levels of sCD14 and sIL-6Ra; and lipid peroxidation in plasma. Inflammasome and mitochondrial status assessments were evaluated by imaging flow cytometry. Mitochondrial superoxide was detected using a flow cytometry-based assay. Monocytes isolated from HC-derived fresh peripheral blood mononuclear cells (PBMCs) were exposed in vitro to SARS-CoV-2. The results showed the following observations for patients with COVID-19: significant changes in circulating monocyte subsets; activation of the systemic inflammasome is associated with disease severity; CD14^high^CD16^−^ monocytes exhibit prominent oxidative stress activity; exacerbated inflammasome activation is associated with mitochondrial dysfunction; aberrant oxidative stress response strongly correlates with robust caspase-1 activation; IL-1β secretion by human monocytes in response to SARS-CoV-2 requires NLR family pyrin domain containing 3 (NLRP3) inflammasome activation and is partially dependent on lipid peroxidation; and excessive oxidative stress response and inflammatory profile persist after short-term recovery [116].

Peluso et al. included in a study and analyzed blood plasma samples donated between 24 April 2020 and 11 February 2021 from four groups of patients, each of them with age- and sex-matched control groups (Ctls, n =15) (the controls having plasma samples donated before the pandemic), divided as follows: (a) group 1 (n = 8) without LC, in convalescence after acute COVID-19 at a distance of at least 4 weeks from infection, without fever, and improvement of symptoms for at least 2 weeks; (b) group 2 (n = 15) with LC, but without neuropsychiatric manifestations (NP); (c) group 3 (n = 15) with LC and with NP symptoms (≤7 symptoms); and d) group 4 (n = 8) with LC and severe NP neuropsychiatric symptoms (>7 symptoms). Depending on how mitochondrial mechanisms modulate the interaction with the virus, it can escape the immunity trap [103], but there is insufficient evidence regarding the exact picture and extent of this mitochondrial intervention in shaping the effects of SARS-CoV-2 infection on the central nervous system (CNS), especially in surviving patients. The authors investigated the SARS-CoV-2 proteins and mitochondrial proteins (MPs) in enriched plasma neuron-derived extracellular vesicles (NDEVs) and astrocyte-derived EVs (ADEVs) in all four groups. Compared with the healthy controls, NDEVs and protein N levels also displayed important differences between sub-groups. Both S1 (RBD) and N proteins were significantly increased in NDEVs than ADEVs of group 3 (LC with NP symptoms), highlighting superior intracellular and feasibly intramitochondrial concentrations in neurons than astrocytes. In this study, the abnormal levels of MPs in plasma ADEVs corresponding to LC groups were limited to mitochondrial ion channels and translocators. Nevertheless, the results must be interpreted with caution because, at this moment, we know very little about the mitophagic pathways used by MPs to enter NDEVs and even less about the same processes concerning ADEVs. Measurements of MPs and viral proteins in plasma NDEVs could be important biomarkers in LC diagnosis and for estimating the effects of new management protocols in the near future [117].

As SARS-CoV-2 penetrates the cell membrane, it also affects the mitochondria of infected cells, thereby triggering altered metabolism, mitophagy, and atypical levels of mitochondrial proteins in extracellular vesicles. To evaluate these parameters as possible biomarkers, Goetzl et al. quantified blood extracellular vesicle SARS-CoV-2 proteins and MPs (mitochondrial proteins, such as MOTS-c, SARM-1, VDAC-1, humanin, LETM-1, and CI-6) by enzyme-linked immunosorbent assays (ELISAs). The study included total extracellular vesicles (TEVs) precipitated from the blood of age- and sex-matched subjects, divided into four groups as follows: G1 (n = 10) = no infection, G2 (n = 16) = acute COVID-19, G3 (n = 30) = post-acute sequelae of COVID-19 or LC, and G4 (n = 8) = post-acute COVID without LC. Intracellular SARS-CoV-2 proteins, that is, higher levels of S1 (receptor binding domain or RBD) protein and N (nucleoprotein), were determined in blood extracellular vesicles (BEVs) in the G2 and G3 groups. Significantly higher levels of several functional MPs were observed in the BEVs of G2 patients who progressed to LC, and after LC installation, these biomarkers indicated the presence of neuropsychiatric manifestations. The persistent state of intracellular SARS-CoV-2 in LC means that therapy for these patients includes cell-permeant agents, that is, antiviral drugs and anti-SARS-CoV-2 nanobodies [118].

The overall picture played by mitochondria in LC is not yet fully understood, especially about pulmonary consequences or long-term complications after infection. Siekacz et al. have sought to investigate the connection between lung problems after COVID-19 and mitochondrial proteins regulating oxidative stress. In total, 80 recovered patients from COVID-19 were randomly selected for this study and divided into two groups: group 1 or (P(+), n = 40) with persistent interstitial lung lesions on computed tomography (CT) and (P(−), n = 40) as the control group without these complications. For all patients, the serum concentrations of mitochondrial regulatory proteins or mitochondrial biomarkers were measured by ELISA. Elevated levels of mitochondrial biomarkers in LC patients with long-term pulmonary sequelae could reveal their feasible role in post-COVID-19 pulmonary pathophysiology. TNF-α could be a valuable and manageable predictor [119].

Gvozdjáková et al. investigated whether a high-altitude environment with fresh air and mountain spa rehabilitation (MR) could improve the mitochondrial bioenergetics of the platelets, accelerate the patient’s recovery, and reduce socio-economic difficulties, according to the recommendations of the European Spa Rehabilitation Association (ESPA). In a pilot study, the researchers examined systematically 14 patients with post-COVID-19 syndrome (G1) compared to 15 healthy subjects (G2 = control group, CG) without medication for chronic diseases and no history of COVID-19. Several sets of measurements (platelet mitochondrial oxidative phosphorylation (OXPHOS) function, coenzyme Q10 level, and thiobarbituric acid-reactive substances (TBARS) concentration) were performed for the two groups: before MR, that is, MR1, and 16–18 days after MR (MR2). It was proven that recovery in a high-altitude environment decreased physical, cognitive, and mental impairment and improved quality of life in patients with post-COVID-19 syndrome [120].

A summary of clinical studies on LC is shown in Table 3.

## 5. Discussions

Long COVID is still an unsolved puzzle for doctors and researchers, present in both children and adults [121]. The effects of SARS-CoV-2 infection, the molecular mechanisms involved in the modulation of intestinal permeability with an impact on autoimmunity [122], as well as the onset and persistence of LC symptoms are not yet fully elucidated, and multiorgan manifestations require multidisciplinary management.

Inflammatory activation through the NF-κB non-canonical pathway and mitochondrial remodeling was experimentally proven in the case of exposure of microvascular endothelial cells of the human brain to SARS-CoV-2 [123].

Inflammation and oxidative stress are among the fundamental mechanisms connected with COVID-19 that are involved in aggravating the illness and sometimes the critical outcomes of patients. Furthermore, the SARS-CoV-2 virus has the capacity to change the intestinal microbiota of patients by decreasing bacteria with probiotic effects, and consequently, the presence of intestinal dysbiosis will exacerbate inflammation and oxidative stress, thus creating a vicious loop that maintains the disorder [124].

Real-world data have shown that post-COVID, there are long-term sequelae of the infection in 31–69% of patients suffering from LC. Among the possible elements identified as responsible is also mitochondrial dysfunction, identified both during the acute infection and after, which could train the LC or PACS. This pathology, recognized as evident today, can manifest itself with different intensities and durations in multiple organ systems, such as post-acute COVID cardiovascular syndrome, post-acute COVID pulmonary syndrome, post-acute COVID neuropsychiatric syndrome, and post-acute COVID multisystem syndrome, despite a mild or asymptomatic initial presentation of the patients. Among the quantifiable risk factors for LC at the time of initial infection are type 2 diabetes, SARS-CoV-2 RNAemia, viremia corresponding to the Epstein–Barr virus, and the presence of specific autoantibodies. In patients with LC and gastrointestinal symptoms, SARS-CoV-2-specific and CMV-specific CD8+ T cells have a distinct evolution during recovery from infection [125].

**Figure 1:**
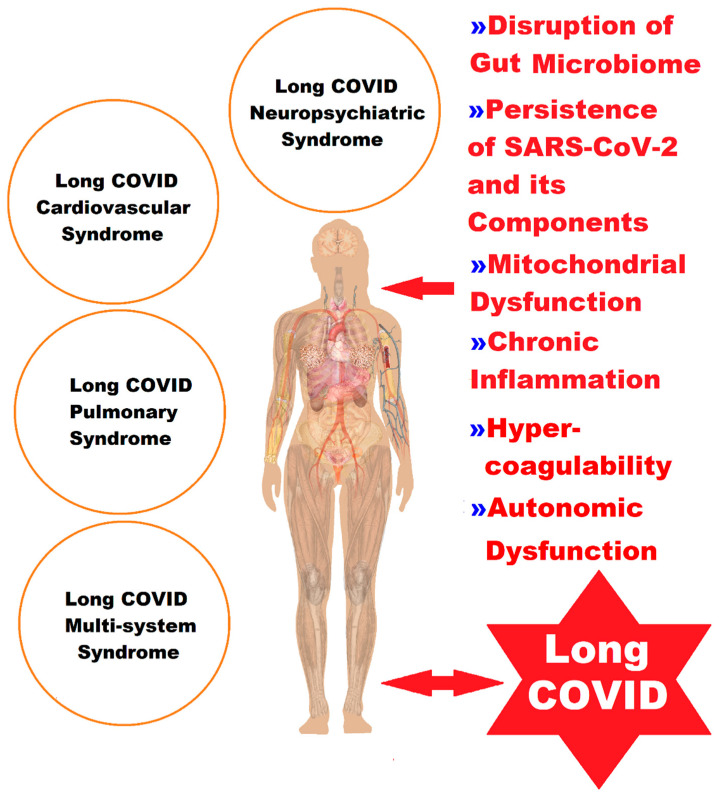
A representation of the complex picture of the clinical manifestations of LC, classified into four syndromes, in correlation with the pathophysiological elements (Figure 1 was imagined and drawn by L.M.A. using Microsoft Paint 3D for Windows 10 and using completely free picture material from SeekPNG.com (accessed on 26 October 2023), for which we are very grateful).

The heterogeneity of LC (Figure 1) has hindered progress in deciphering all the pathophysiological mechanisms, and therefore, the approach must be multidisciplinary, with a special focus not only on symptomatic management but also on addressing the underlying health problems of the patients.

The old-fashioned depiction of mitochondria only as the power plants of living cells should have long since disappeared. Mitochondria are essential organelles in living cells and are maternally inherited and full of dynamism; they have sophisticated intrinsic mechanisms of energy production and transduction, biosynthesis of living metabolites, and finally, signaling and active transduction of biological information at a distance. Supported by real data from the latest research and discoveries in recent years, mitochondria must be reimagined as an important processor of the cell, which, together with the nucleus and other organelles, constitute the mitochondrial information processing system that performs operations on the information inside and transmits it remotely to the outside of the cell. As the model is imagined today, mitochondria are able: (a) to perceive and react to both endogenous and external stimuli through structural and functional rearrangement, (b) to unify all information through dynamic channels and paths specific to multinetwork physical interactions and through diffusion processes, and (c) to emit output signals that not only fine-tune the functions of other organelles but also permanently regulate physiological processes at the level of the cell, organ, and organism. The multitude and diversity of input-to-output information is taken over and transduced by mitochondria into metabolic, biochemical, and neuroendocrine signals, as well as other local or systemic signals, which improve the body’s adaptation [126].

Starting from this model, mitochondria, as bioenergetic, biosynthesis, and signaling organelles, transduce and modulate physiological adaptations and responses to environmental stress, which could modify mitochondrial homeostasis [126,127], such as changes in the intestinal microbiome in SARS-CoV-2 infection, with repercussions regarding OXPHOS, ROS, free radicals, and others, as well as mitochondrial biogenesis processes. The fusion and fission that are the basis of mitochondrial biogenesis, and the mitophagy of damaged mitochondria, could have a positive dynamic balance or could illustrate mitochondrial dysfunction.

Intestinal microbiota, by participating in immune defense functions, metabolic adjustment, and modulating pathways on the gut–lung–brain axis, has an essential role in maintaining health. Metagenomic sequencing studies of stool samples demonstrated a direct correlation between the intestinal reduction in bacteria such as *Faecalibacterium prausnitzii*, *Bifidobacterium adolescentis*, and *Eubacterium rectale*, with the persistence of clinical symptoms of chronic fatigue and brain fog, and an increased percentage of *Actinomyces viscosus*, *Clostridium hathawayis*, *Streptococcus*, *Ruminococcus gnavus*, and *Bacteroides vulgatus* in patients infected with COVID-19 and in those with LC. Recent publications analyzed in this review show that the respiratory and neuropsychiatric symptoms of LC are directly proportional to the presence of intestinal nosocomial pathogens such as *Clostridium innocuum* and *Actinomyces naeslundii* and inversely proportional to the reduction in *Bifidobacterium pseudocatenulatum* and *Faecalibacterium prausnitzii*—butyrate-producing bacteria [89,92].

On the gut–lung axis, respiratory dysfunction was correlated with the reduction in good butyrate-producing bacteria, probably through the removal of microorganisms with an immunomodulatory effect, a phenomenon that would be the basis of the severity of the clinical manifestations, the persistent inflammatory processes, and the late complications of COVID-19.

The complex interplay between infection, dysbiosis, and inflammation at a molecular and cellular level could better guide scientists and physicians to advance new lines of medical action to avoid the post-acute sequelae of SARS-CoV-2 infection or LC [121].

**Figure 2:**
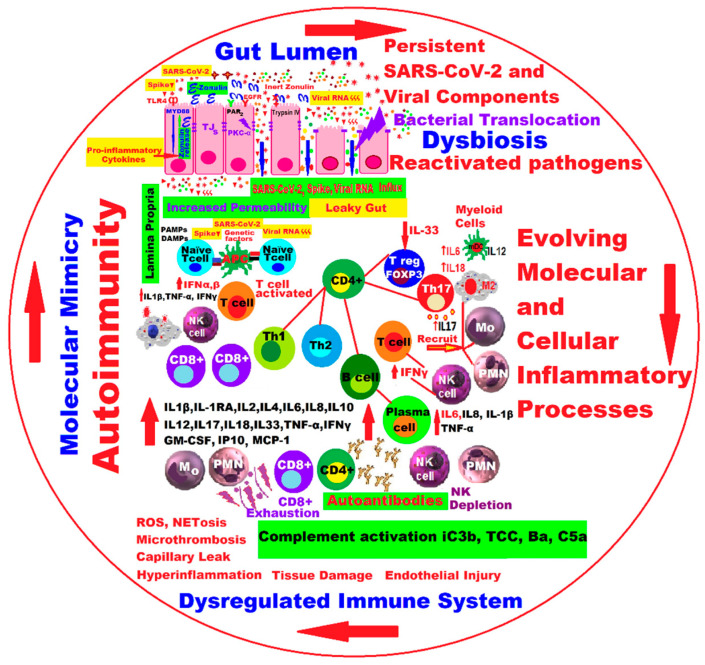
New insights and hypotheses regarding the molecular and cellular processes in the vicious circle that would trigger the pathophysiological manifestations in LC. The arrows indicate the closing of the dangerous circle that self-maintains and amplifies the inflammatory phenomena (Figure 2 was imagined and drawn by L.M.A. using Microsoft Paint 3D for Windows 10 being modified and drawn by the same main author, L.M.A. [121]).

**Figure 3:**
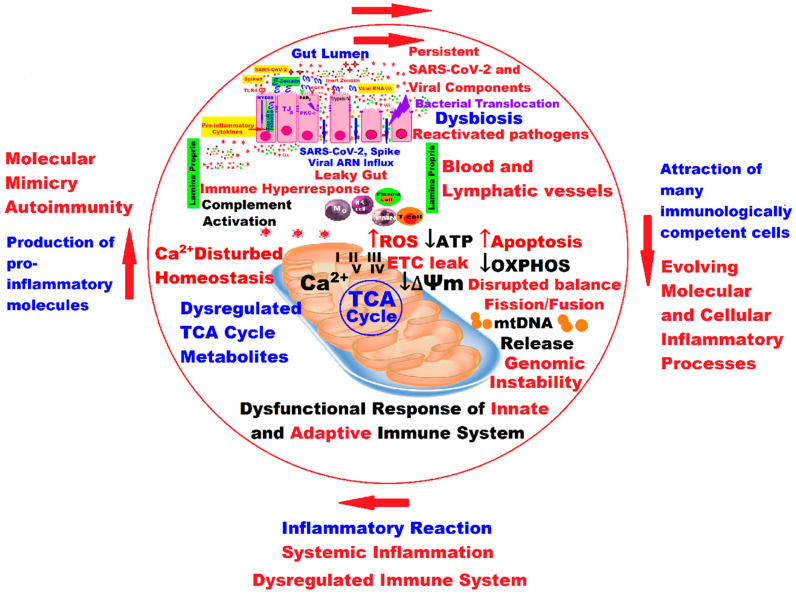
Mitochondrial dysfunction in LC correlated with the synergistic interaction between infection, gut dysbiosis, systemic inflammation, dysregulated immune system, molecular mimicry, and auto-immunity, generating a vicious self-sustained circle and reflecting molecular and cellular processes from “leaky gut” to “leaky ETC” in a quantum leap. The arrows indicate the closing of the dangerous circle that self-maintains and amplifies the inflammatory phenomena (Figure 3 was imagined and drawn by L.M.A. using Microsoft Paint 3D for Windows 10 and using completely free picture material from SeekPNG.com (accessed on 26 October 2023), for which we are very grateful), being modified after Figure 2 [121]).

Dysregulation with multiple faces of mitochondria can trigger the onset and progression of the LC, starting from the critical molecular machinery that regulates the functioning of the mitochondria, which in LC is reflected by the loss of mitochondrial membrane potential (see also Table 3), “leaky ETC”, a decrease in adenosine triphosphate (ATP) production, OXPHOS, and so on (Figure 2 and Figure 3), practically from “leaky gut” to “leaky ETC” into a quantum leap!

Another hypothesis would be complement activation in a severe SARS-CoV-2 infection. In this sense, the chronic or persistent inflammatory response associated with the deregulation of the complement system, microthrombosis phenomena, and endothelial dysfunction [128] could trigger various manifestations in LC, according to some recent studies in which they were identified as reliable biomarkers (fractions Ba, iC3b, C5a, and TCC of the complement system) for the diagnosis of LC [129].

Serum testing of complement biomarkers, together with serotonin and cortisol levels [130], as well as the loss of leukocyte ΔΨm, could be a solution for a precise diagnosis of LC and future individualized therapies.

The last scientific research highlighted the essential signaling functions of mitochondria, an extremely important role unknown until now as “signaling molecules” of the intermediate products of the tricarboxylic acid cycle (TCA cycle), with significant functions controlling chromatin modifications, DNA methylation, hypoxic response, and immunity. The TCA cycle is a signaling hub, and the TCA cycle and OXPHOS are tightly coordinated [131].

Acting as a coenzyme, nicotinamide adenine dinucleotide (NAD+) plays pivotal roles in energy metabolism pathways, including glycolysis, the TCA cycle, OXPHOS, FAO, and ethanol metabolism [132,133].

The role of NAD+ in metabolic stress is essential. If antioxidant processes are impaired, ROS can accumulate and cause DNA damage, leading to decreased NAD+ levels and increased inflammation that becomes chronic by triggering pro-inflammatory transcription factors with negative effects on the central nervous system’s health due to intracellular signaling cascades that promote the expression of pro-inflammatory genes. The whole picture of the molecular and cellular mechanisms involved in LC is under investigation and remains insufficiently elucidated, but there is more and more obvious evidence that the damage to the NAD + metabolome and, consequently, the mitochondrial dysfunction that follows through the integration of the virus genome, which decreases NAD+ availability for host metabolism and, in the end, causes cell death, can trigger elements of the pathogenesis of LC. A complex interplay exists between increased oxidative stress, inflammation, and mitochondrial dysfunction. Thus, the depletion of NAD+ mediated by viral replication could trigger the subsequent abnormalities present in the LC. The authors’ conclusion is that intravenous administration of NAD+ could be a new way of managing LC [134].

Although human clinical trials are still at an early stage in modulating NAD+ concentrations or the activity of NAD+-consuming enzymes, it is hoped that such medical interventions will be feasible in the COVID era to protect against the progression of various pathologies after COVID-19 and future viral infections. NAD+ metabolism can regulate the innate and adaptive immune systems and contribute to inflammatory responses, not only as a critical cofactor in energy metabolism but also as a cosubstrate for non-redox NAD+-dependent enzymes. Thus, research on the modulation of NAD+ bioavailability may reshape the evolution of immune diseases and hold great future translational potential for new therapies for inflammatory pathologies such as SARS-CoV-2 infection. Meanwhile, dietary precursors such as NR (nicotinamide riboside) and NMN (nicotinamide mononucleotide) can promote NAD+ biosynthesis and increase its intracellular levels, opening avenues for new strategies and future perspectives through their beneficial physiological effects and their interaction with the gut microbiota, which reverse physiological decline and prevent disease through nutrition and food science research [135,136,137,138,139].

Obesity, diabetes, and the ongoing effects of LC worldwide are increasing and putting pressure on health systems. It is assumed that in the near future, the burden of cardiovascular disease (CVD), that is, morbidity and mortality due to CVD, especially on a large scale, will increase. Ca^2+^ influx is primarily through the L-type Ca^2+^ channel, and acute, severe changes in Ca^2+^ influx through this channel could lead to arrhythmia and even sudden cardiac arrest. Meanwhile, chronic increases in intracellular Ca^2+^ concentrations could lead to pathological cardiac hypertrophy and heart failure. Elucidating the role of the L-type calcium channel in excitability and energetics in the heart was carried out by a team of Australian researchers. Over the past two decades, Hool and her team have applied multiple disciplines simultaneously to investigate channel control under physiological and pathological conditions, ranging from the molecular level to in vivo CRISPR mutant experimental prototypes. Using single-channel analysis, they revealed the direct molecular mechanisms for driving the channel, such as the critical serine involved in the fight-or-flight response and the cysteine responsible for altered function during oxidative stress. Researchers have shown in a premiere that mitochondrial function and cellular energy can be changed by beats triggered by protein movement in the cytoskeletal structure during L-type Ca^2+^ channel activation. So, the cytoskeletal protein network of the cardiac myocytes modulates the L-type Ca^2+^ channel, which, in turn, regulates mitochondrial function, opening other perspectives to test new experiments on possible future therapies for preventing cardiomyopathies [140].

Advances in scientific applications of gene editing to manipulate the molecular basis of the L-type Ca^2+^ channel will increase mastery of the structure–function relationship of this vital channel. As the cytoskeleton has been shown to play an essential role in the distribution and regulation of mitochondria, targeting L-type Ca^2+^ currents to prevent mitochondrial dysfunction could be a novel pathway to outline individual therapies for preventing and treating the associated cardiac symptoms in LC.

SARS-CoV-2 uses host cells to replicate and spread. SARS-CoV-2 viral proteins bind to host mitochondrial proteins, likely inhibiting OXPHOS and stimulating glycolysis. In a very recently published study, researchers investigated human nasopharyngeal samples and autopsy tissues from patients with COVID-19, as well as tissues from hamsters and mice infected with SARS-CoV-2. The ability of the virus to stop the expression of not only nuclear-encoded mitochondrial genes but also mitochondrial genes has been demonstrated, a process that dramatically influences and modifies the functions of host cell mitochondria. The virus blocked the transcription of a subset of nuclear DNA (nDNA)-encoded mitochondrial OXPHOS genes, induced the expression of microRNA 2392, and activated HIF-1α to induce glycolysis. In response, host cells fight back and activate the immune defense system, including the integrated stress response and the corresponding mitochondrial gene expression. However, the persistence of mitochondrial dysfunction and its chronicity can induce serious sequelae of COVID-19, leading to the failure of many organs. Even if the immune system manages to eliminate the virus from the lung and the function of the mitochondria is fully recovered in the lung, in other organs such as the heart, and, to a lesser extent, kidneys, liver, and lymph nodes, the mitochondria remain dysfunctional (nDNA mitochondrial gene expression remained suppressed, whereas mitochondrial DNA (mtDNA or mDNA) transcription was induced and host-immune defense pathways were activated), which can generate severe conditions [141].

## 6. Conclusions

This review highlights the key role of mitochondrial dysfunction in LC, related to the complicated reciprocal action and reaction between infection, gut dysbiosis, dysfunctional mitochondria, and systemic inflammation generated in a vicious circle, reflecting the molecular and cellular processes from the “leaky gut” to the “leaky ETC” into a quantum leap.

The imbalance of the intestinal microbiome with the reduction in commensal bacteria producing beneficial SCFAs that support the integrity of the intestinal mucosa competes for the occurrence of inflammatory phenomena, the hyperactivation of the immune system, mitochondrial dysfunction, and the increase in the level of autoantibodies.

Stressful activities during the COVID-19 pandemic produced intestinal dysbiosis for a period of several months, with negative effects on mental health in patients infected with SARS-CoV-2.

Ectopically colonized bacteria from the intestine in the oral cavity in LC patients and digestive symptoms correlated with harmful metabolites in the serum could be the basis of the pathogenic mechanisms in this clinical pathology.

The persistence of SARS-CoV-2 and its remnants, together with dysfunctional mitochondria in the heart, kidney, liver, lymph nodes, and brain, could be an explanation for LC and needs to be further studied.

To prevent a decline in the quality of life among post-COVID-19 patients, follow-up and assistance through individualized health programs are essential.

## Figures and Tables

**Table 1:** The multifactorial etiology of the pathophysiology of LC.

Host Conditions	Viral Agents	Downstream Effects
Age Sex Ethnicity Genetic factors Metabolic/endocrine diseases Chronic inflammation Immunological imbalances/autoimmune diseases	Occult persistence of the SARS-CoV-2 virus Persistence of SARS-CoV-2 viral components Reactivation of latent viruses [(Epstein–Barr virus (EBV), Cytomegalovirus (CMV), human immunodeficiency virus (HIV), herpes simplex virus 1, human herpesvirus 6 (HHV-6), and human herpesvirus 7 (HHV-7)]	Grade of lesions from primary acute SARS-CoV-2 infection Vascular endothelial abnormalities Microclots Thromboses Dysfunctional neurological signaling Reduction in tissue oxygen/hypoxia Disruption of the intestinal microbiome

**Table 2:** Dysbiosis of the gut microbiota and its interrelationship with LC.

Reference	Patients COVID-19/LC	Measured Parameters				Conclusions
		Fecal Samples	Respiratory Tract Samples	Increased Opportunistic Pathogens	Reduced Microbial Biodiversity	
[92] Liu, Q. et al., 2022; https://doi.org/10.1136/gutjnl-2021-325989	G1: 106 patients with LC. G2: 68 non-COVID-19 patients	258 stool samples	-	Yes	Yes	Evidence of gut microbiome composition changes in LC. Could its modulation be useful in LC recovery?
[93] Mazzarelli, A. et al., 2022; https://doi.org/10.3389/fmicb.2022.1049215	97 patients—SARS-CoV-2 infection	97 rectal swabs	-	Yes	Yes	The gut microbiota profile varies with the severity of the SARS-CoV-2 infection and may be a prognostic biomarker.
[96] Gao, F. et al., 2022; https://doi.org/10.1016/j.jad.2022.02.024	G1 = 71 FHW-treated patients with COVID-19. G2 = 104 SHWs who treated non-infected patients with COVID-19.	Bacterial genomic DNA was extracted and analyzed.	-	Yes	Yes	Stress-triggered intestinal dysbiosis in FHWs was persistent for at least 6 months. Neuropsychiatric symptoms in FHWs were correlated directly with the intestinal microbiome.
[97] Vestad, B. et al., 2022; https://doi.org/10.1111/joim.13458	Randomized trial of 181 patients with COVID-19, divided into 3 subgroups.	S1 = Rectal swab material and 16S rRNA gene sequencing. S2 = lung function tests. S3 = rectal swabs and pulmonary function tests.	Pulmonary function tests.	Yes	Yes	Respiratory dysfunction in LC could be correlated with an altered gut microbiome and elevated LBP levels. Possible involvement of the gut–lung axis in LC.
[98] Liu, Q. et al., 2022; https://doi.org/10.1038/s41467-022-34535-8	Cross-sectional and prospective study on a cohort of 133 COVID-19 patients followed for up to 6 months.	Integrated analysis: 296 fecal metagenomes. 79 fecal metabolomics. 1378 viral loads in respiratory tract samples.	Viral load in 1378 respiratory tract samples (sputum and nasopharyngeal sample).	Yes	Yes	Host phenotype and multikingdom microbiota profile could be prognostic factors for COVID-19.
[99] Tkacheva, O.N. et al., 2023; https://doi.org/10.3390/microorganisms11041036	178 patients with post-COVID-19 and contacts for SARS-CoV-2 but without infection.	Fecal samples	-	Yes	Yes	Three months after infection with SARS-CoV-2, the intestinal microbiota was restored, and no significant differences in its composition were found. Novel strategies for microbiome-tailored disease prevention and treatment are needed.
[100] Caio R. et al., 2023; https://doi.org/10.26355/mhd_20233_818	46 patients aged between 30 and 95, hospitalized with COVID-19, were grouped by clinical severity (i.e., non-critical or critical), type of hospitalization (non-intensive care or intensive therapy unit), and outcome.	Stool samples were analyzed by shotgun metagenomic sequencing.	-	Yes	Yes	Intestinal dysbiosis could underlie disease severity, persistent inflammation, and late complications in LC.
[101] Zhang, D. et al., 2023; https://doi.org/10.3346/jkms.2023.38.e120	187 RPs, among them, 84 (44.9%) reported LC one year after discharge.	In 130 RPs and 32 HCs: Stool samples collection and 16s rRNA sequencing.	-	Yes	Significantly reduced bacterial diversities and a lower relative abundance of SCFAs.	SCFAs and SCFA-producing commensal bacteria may delay recovery and sustain the persistence of LC.
[102] Zhang, D. et al., 2023; https://doi.org/10.1186/s12916-023-02972-x	Prospectively analyzed oral, fecal, and serum samples from 983 antibiotic-naïve subjects with mild COVID-19 were monitored for 3 months after discharge.	45 fecal and saliva samples and 25 matched serum samples were collected from patients who had LC with digestive symptoms, compared to HCs.	8 saliva and fecal samples were collected from patients with LC but without digestive symptoms.	Yes	Yes	Patients with digestive symptoms of LC after mild forms of COVID-19 may have an ectopic colonization of the oral microbiome with gut microbes and a disturbance of serum metabolites.

**Table 3:** Clinical studies, molecular targets, and possible implications in LC.

No	References	Study Design	Targets/Trial Protocol/Main Parameters Measured	Brief Results	Conclusions
1.	[109] Grossini, E. et al., 2021. https://doi.org/10.3389/fphys.2021.707587	60 subjects mostly women (mean age 84 years), 12 years older than men, admitted to a LTC facility. All without cognitive impairment.	Plasma markers of lipidic peroxidation: thiobarbituric acid reactive substances (TBARS) release, 8-hydroxy 2 deoxyguanosine (8 OH-2dG), 8-isoprostanes, superoxide dismutase (SOD) activity, glutathione (GSH), and 25(OH) vitamin D. Thymosin β4 (human TMS β4). Cell viability, mitochondrial membrane potential, and ROS on HUVEC.	TBARS, 8 OH-2dG, and 8-isoprostanes exhibited an “oxidative” plasma status. The antioxidant system was well preserved. Vitamin D and GSH were within the physiological range. SOD activity was about 51%. HUVEC treatment with plasma has reduced cell viability by about 60% and increased ROS by about 80% compared to untreated HUVEC.	Assessment of mitochondrial function in the elderly hospitalized in LTC facilities is essential for estimating susceptibility to COVID-19 and identifying patients at high “risk” for the development of infections.
2.	[110] Levy, D. et al., 2022; https://doi.org/10.3390/nu14040912	139 patients who survived after COVID-19 and admitted to the ICU.	Sarcopenia and weight evolution at 3 (M3) and 6(M6) months after ICU discharge.	At M3: Sarcopenia (n = 22), weight decrease > 5% (n = 13). At M6: Persistent sarcopenia: n = 6. Recovering from sarcopenia: n = 16.	The persistence of sarcopenia was associated with female sex, older age, and more severe baseline sarcopenia. In a holistic approach, sarcopenia is reversible through individualized nutritional programs and personalized physical rehabilitation.
3.	[111] Ghanem, J. et al., 2022; https://doi.org/10.3390/nu14153027	37 patients hospitalized for a severe SARS-CoV-2 infection.	Long-term evaluation of autonomy, malnutrition, and LC symptoms.	An important decrease in autonomy is associated with malnutrition after ICU hospitalization. Beneficial effects of personalized rehabilitation.	6 months after discharge: 20% are still without full autonomy; 70% are still with chronic fatigue. Need for personalized and persistent follow-up.
4.	[112] Guntur, V.P. et al., 2022; https://doi.org/10.3390/metabo12111026	Plasma samples from 75 patients divided into 3 groups: G1: LC. G2: fully recovered. G3: healthy controls.	Mass spectrometry-based untargeted metabolomics.	Higher levels of fatty acid metabolites; lower levels of mono-, di-, and tri-carboxylates; and depletion of tryptophan in plasma samples of patients with LC (G1).	The need for therapeutic intervention to restore mitochondrial fat-burning capacity in LC.
5.	[113] Díaz-Resendiz, K. et al., 2022; https://doi.org/10.1002/JLB.3MA0322-279RRR	Human plasma study with 4 groups: HC, C-19, R1, and R2.	ΔΨm measured in human leucocytes for all 4 groups.	ΔΨm was decreased in all three groups compared to healthy controls, even 11 months post-infection; a sex-associated response.	The loss of ΔΨm could indicate a susceptibility to developing LC.
6.	[114] Díaz-Resendiz, K.J.G. et al., 2022; https://doi.org/10.3390/md20020099	76 subjects, divided into different groups, were administered Fucoidan. Phase 1: HC (n = 24) C-19 (n = 31) R1 (n = 21). Phase 2: HC (n = 19) R2 (n = 19).	Ex-vivo fucoidan treatment in HPBMCs. ∆Ψm measurements.	COVID-19 induces an elevated inflammatory/ oxidative state, mitochondrial dysfunction, and ∆Ψm loss.	Fucoidan may constitute a potential treatment to prevent LC, with mitochondria as a therapeutic target to restore homeostasis and ∆Ψm.
7.	[115] Pozzi, A., 2022. https://doi.org/10.3389/fphys.2021.805005	RNA samples extracted from PBMC in patients recovering from COVID-19.	Expression of canonical and non-canonical genes encoded on the mitochondrial genome.	Only some non-canonical mitochondrial genes are disrupted by COVID-19, being limited to mt-sRNAs, without altering the overall mitochondrial transcription.	Further studies on the role of mt-sRNAs in LC are required.
8.	[116] Lage, S.L. et al., 2022; https://doi.org/10.3389/fimmu.2021.799558	47 COVID-19 patients, enrolled from March 2020 to August 2020, divided into mild (n = 31) and moderate-severe (n = 16) groups.	Plasma biomarkers. Inflammasome and mitochondrial status. Lipid peroxidation. Intracellular GSH levels. Mitochondrial superoxide. Circulating monocyte subsets.	↑↑CD14^high^ CD16^−^ classical monocytes compared to HCs. ↑Inflammasome activation. ↑Oxidative stress/NLRP3 signaling pathway. Target therapy to mitigate early hyperinflammation and LC outcome.	Sustained deregulated oxidative stress and inflammasome activation in monocytes after short-term recovery support one of the current hypotheses that LC is driven by persistent pathological inflammation and suggest the pathways involved as potential targets for the management of LC.
9.	[117] Peluso, M.J. et al., 2022; https://doi.org/10.1002/ana.26350	Human plasma study with 4 groups, relative to controls. G1: post-COVID, without LC, G2: LC without NP, G3: LC with NP, and G4: LC with severe NP.	Measurements of SARS-CoV-2 proteins and MPs in NDEVs and ADEVs.	S1 and N proteins were increased in all LC subgroups compared to controls; N concentrations were higher in LC with NP.	Development of new biomarkers and a faster effective technology to identify MPs or SARS-CoV-2′s protein abnormalities in NDEVs or ADEVs during acute infection to accurately predict the risk of developing LC.
10.	[118] Goetzl, E.J. et al., 2023; https://doi.org/10.1016/j.amjmed.2023.03.026	4 study groups: G1= no infection, G2= acute infection, G3 = LC, and G4= post-acute COVID without LC.	Measurements of plasma TEVs proteins in all 4 groups.	For SARS-CoV-2 S1 (RBD) and N: - confirmation of the intracellular presence of the virus. - detection of a specific strain of SARS-CoV-2. For functional MP altered by SARS-CoV-2 in G3 (or LC): ↓MOTS-c, VDAC-1, and humanin. ↑SARM-1 in G2 that progressed to LC.	Management with anti-viral drugs. Abnormal levels of humanin, MOTS-c, and SARM-1 in LC predict neuropsychiatric symptoms.
11.	[119] Siekacz, K. et al., 2023; https://doi.org/10.3390/jcm12134253	80 patients post-COVID-19 divided into two groups: 1. (P(+), n = 40) with persistent interstitial lung lesions on CT. 2. (P(−), n = 40) without lung lesions on CT.	Mitochondrial biomarkers by (ELISA).	P(+) compared to P(−): ↑PTEN-induced kinase 1 (PINK1). ↑Dynamin-1-like protein (DNM1L). ↑Mitofusin-2 (MFN2). ↑Chemokine ligand 18 (PARC, CCL18). ↑IL-6 and ↑ tumor necrosis factor-alpha (TNF-α). ↓Interferon alpha (IFN-α). In P(+) patients: correlations between: - advanced glycation end product (sRAGE) and TNF-α - between DNM1L and IFN-α.	SARS-CoV-2 could trigger mitochondrial dysfunction and chronic inflammation by deregulating PINK1, DNM1L, and MFN2. ↑↑ CLL18, TNF-α, and IL-6 could support long-term pulmonary complications in LC. TNF-α = a potential predictor.
12.	[120] Gvozdjáková, A. et al., 2023; https://doi.org/10.1007/s11356-022-22949-2	2 groups: G1 = 14 LC patients compared to 15 healthy subjects (G2= CG), before and after MR.	Functional capacity of the lungs. Questionnaire for clinical symptoms before and after MR. Blood count and biochemical parameters. CoQ10 and TBARS. Mitochondrial bioenergetics in platelets. Citrate synthase as a mitochondrial marker.	Important adjustment of clinical symptoms, lung function, and regeneration of platelet mitochondrial metabolism after MR.	High-altitude SPA rehabilitation accelerates post-COVID recovery by improving mitochondrial bioenergetics.

## Data Availability

The literature used for this article is available from the first author.

## Abbreviations

**ACE2**	angiotensin-converting enzyme 2
**ADEVs**	astrocyte-derived EVs
**AG-GIR**	Autonomie Gérontologie Groupes Iso-Ressources
**ATP**	adenosine triphosphate
**Ba**	fragment of complement factor B that results from activation of the alternative pathway
**B cell**	B lymphocytes
**BEVs**	blood extracellular vesicles
**C5a**	complement component 5a; protein fragment released from the cleavage of complement component C5 by protease C5-convertase into C5a and C5b fragments
**Ca^2+^**	calcium ions
**CMV**	cytomegalovirus
**CCL18**	chemokine ligand 18 (PARC).
**COVID-19**	coronavirus disease 2019
**CNS**	central nervous system
**CRM**	rehabilitation center
**CRP**	C-reactive protein
**CX3CR 3**	CX3C motif chemokine receptor 3
**DAMP**	damage-associated molecular pattern
**DNA**	deoxyribonucleic acid
**DNM1L**	dynamin-1-like protein
**ELISA**	enzyme-linked immunosorbent assays
**EV**	extracellular vesicle
**ESPA**	European Spa Rehabilitation Association
**ETC**	electron transport chain
**EWGSOP2**	European Working Group on Sarcopenia in Older People
**FHWs**	first-line healthcare workers
**HBMECs**	human brain microvascular endothelial cells
**HCs**	healthy controls
**HIV**	human immunodeficiency virus
**HLA**	human leukocyte antigen
**HHV-6**	human herpesvirus 6
**HHV-7**	human herpesvirus 7
**HPBMC**	human peripheral blood mononuclear cells
**HUVEC**	human umbilical vascular endothelial cells
**GLIM**	Global Leadership Initiative on Malnutrition
**GSH**	glutathione
**iC3b**	protein fragment part of the complement system, produced when complement factor I cleaves C3b
**Ig**	immunoglobulin
**IgA**	immunoglobulin A
**IgG**	immunoglobulin G
**IgM**	immunoglobulin M
**ICAM**	intercellular adhesion molecule
**ICU**	intensive care unit
**IFN**	interferon
**IFN-α**	interferon alpha
**IP-10**	IFN-gamma-inducible protein 10 (IP-10, CXCL10)
**IFN-γ**	interferon-gamma
**IL-1, IL-6, IL-8, IL-10, IL-12, IL-12p70, IL-15**	interleukin 1, 6, 8, 10, 12, 12p70, 15, 18, 27
**IL-1α**	interleukin 1α
**IL-1β**	interleukin 1β
**ITIM**	immunoreceptor tyrosine-based inhibitory motif
**LC**	long COVID
**LBP**	lipopolysaccharide-binding protein
**LTC**	long-term care
**MAVS**	mitochondrial antiviral-signaling protein
**ΔΨm**	mitochondrial membrane potential
**MPs**	mitochondrial proteins
**MFN2**	mitofusin-2
**Mo**	monocyte
**MR**	mountain spa rehabilitation
**ME/CFS**	myalgic encephalomyelitis/chronic fatigue syndrome
**mtDAMPs**	damage-associated molecular patterns
**mtDNA or mDNA**	mitochondrial DNA
**N**	nucleoprotein
**NAD+**	nicotinamide adenine dinucleotide
**NK**	natural killer
**NDEVs**	neuron-derived extracellular vesicles
**NIHR**	National Institute for Health Research
**NIH**	National Institutes of Health
**NF-κB**	nuclear factor kappa-light-chain-enhancer of activated B cells
**NLRP3**	NLR family pyrin domain containing 3
**NMN**	nicotinamide mononucleotide
**NP**	neuropsychiatric manifestations
**NR**	nicotinamide riboside
**OXPHOS**	oxidative phosphorylation or electron transport-linked phosphorylation
**PAMP**	pathogen-associated molecular pattern
**PASC**	post-acute sequelae of COVID-19
**PBMC**	peripheral blood mononuclear cells
**PCR**	polymerase chain reaction
**PC**	post-COVID syndrome
**PCC**	post-COVID-19 condition
**PD-1**	programmed cell death protein 1
**PD-L1**	programmed cell death protein ligand 1
**PD-L2**	programmed cell death protein ligand 2
**PELORA**	PEnalized logistic regression analysis
**PMN**	polymorphonuclear
**PINK1**	PTEN-induced kinase 1
**RPs**	recovered patients
**RBD**	receptor binding domain
**RT-qPCR**	real-time quantitative reverse transcription PCR
**ROS**	reactive oxygen species
**RBD**	receptor binding domain
**T cell**	T lymphocytes
**Tregs**	regulatory T lymphocytes
**RAS**	renin–angiotensin system
**RT-PCR**	reverse transcriptase polymerase chain reaction
**RNA**	ribonucleic acid
**S**	spike protein
**sRAGE**	advanced glycation end product
**SARS**	severe acute respiratory syndrome
**SAA**	serum amyloid A
**SARS-CoV-2**	severe acute respiratory syndrome coronavirus 2
**SCFAs**	short-chain fatty acids
**SHWs**	second-line healthcare workers
**SOD**	superoxide dismutase
**TBARS**	thiobarbituric acid-reactive substances
**TCA cycle**	tricarboxylic acid cycle
**TCC**	terminal complement complex or membrane attack complex (MAC)
**TIGIT**	T-cell immunoreceptor with immunoglobulin and immunoreceptor tyrosine-based inhibitory motif domains
**TEVs**	total extracellular vesicles
**Th1**	T-helper 1 or T-helper type 1
**Th2**	T-helper 2 or T-helper type 2
**Th17**	T-helper 17 or T-helper type 17
**TJs**	tight junctions
**TLR2**	toll-like receptor 2
**TLR4**	toll-like receptor 4
**TLR**	toll-like receptors
**TLR7**	toll-like receptor 7
**TMPRSS2**	transmembrane serine protease 2
**TMPRSS4**	transmembrane serine protease 4
**TNF-α**	tumor necrosis factor alpha
**TGF-β**	transforming growth factor-β
**TKI**	tyrosine kinase inhibitors
**US**	United States
**FDA**	U.S. Food and Drug Administration
**UK**	United Kingdom
**UKRI**	UK Research and Innovation
**VCAM-1**	vascular cell adhesion molecule 1
**WHO**	World Health Organization
**Increased**	↑
**Decreased**	↓
**Present**	+
**Absent/Missing**	-
